# Comment on “Reporting of morbidity data in general surgery: a one-year cross-sectional analysis of the six journals with the highest impact factor”

**DOI:** 10.1097/JS9.0000000000004786

**Published:** 2026-01-13

**Authors:** Tingting Wang

**Affiliations:** Reproductive Medicine Center, Hainan Women and Children’s Medical Center, Haikou, China

*To the Editor,*

Gorini *et al* provide a timely audit of postoperative morbidity reporting in six high-impact general surgery journals (2022; 249 studies), showing that even in elite outlets, complication reporting often lacks the methodological transparency needed for reproducibility and benchmarking[1]. This commentary was prepared in line with the TITAN 2025 guideline to promote transparent and accountable reporting[2].

Three deficiencies highlighted by the authors are particularly consequential. First, data provenance is frequently unclear: most studies did not consistently state where morbidity data came from or who adjudicated and recorded complications, making ascertainment bias impossible to judge^1^. Second, post-discharge capture remains a blind spot – although follow-up was often mentioned, follow-up methods were rarely described, and explicit confirmation that complications were collected during follow-up was essentially absent[1]. Given that many clinically meaningful complications occur after discharge, incomplete surveillance systematically underestimates morbidity and distorts inter-study comparisons. Third, although standardized grading was common, the authors document extensive heterogeneity and modification of Clavien–Dindo reporting, including selective thresholds (e.g., only ≥grade III), which risks “hiding” minor complications that still drive costs and patient experience[1]. This is notable because Clavien–Dindo was explicitly designed to be used without ad hoc modification to preserve comparability^[3,4]^.

Importantly, the problems identified are not new; they echo the classic Annals of Surgery work by Martin *et al*, which proposed 10 core elements for rigorous complication reporting more than two decades ago[5]. The persistence of these gaps – despite near-universal endorsement of reporting guidelines – suggests that voluntary adherence is insufficient. Gorini *et al* therefore make a persuasive case for moving from “recommended” to “required” morbidity reporting items. Their six core elements (time window, data sources, data collectors, standardized grading, follow-up methods, readmission reporting) provide an immediately implementable minimum dataset[1].

A practical next step would be journal-level enforcement: a short, mandatory “morbidity methods” box (analogous to CONSORT/STROBE check items, and consistent with STROCSS updates) could standardize complication capture and substantially improve meta-analytic usability^[6,7]^. If adopted, this would reduce outcome-reporting variability, strengthen quality-improvement claims, and improve the credibility of technique comparisons across surgical research.

## Data Availability

No data were used in this Letter to the Editor.
